# The increasing role of artificial intelligence in radiation oncology: how should we navigate it?

**DOI:** 10.1007/s00066-025-02381-4

**Published:** 2025-02-19

**Authors:** Florian Putz, Rainer Fietkau

**Affiliations:** 1 Department of Radiation Oncology, Universitätsklinikum Erlangen, Friedrich-Alexander-Universität Erlangen-Nürnberg, Universitaetsstraße 27, 91054 Erlangen, Germany; 2 Bavarian Cancer Research Center (BZKF), Munich, Germany; 3 Comprehensive Cancer Center Erlangen-EMN (CCC ER-EMN), Erlangen, Germany

## Why the role of AI in radiation oncology is set to grow

As illustrated by this special issue, artificial intelligence (AI) technology [1] has a potential impact on all aspects of radiation oncology, ranging from automated treatment planning [2], adaptive radiotherapy, and IGRT [3, 4], to clinical decision support, personalization [5], and improvement of patients’ experience and quality of life [6]. Radiation oncology may be impacted by AI more significantly than any other therapeutic medical field, as radiotherapy treatment planning and delivery are already fully computer based, thus greatly facilitating the introduction of AI technology. Given the increasing role of AI in radiation oncology, it is important to discuss potential implications and the optimal strategy for our field. In particular, the potential of AI to automate virtually all steps of the treatment planning chain is a double-edged sword that brings about some of the greatest benefits but also affects the unique competency of radiation oncology. Moreover, we must not overlook the clinical aspects of radiation oncology, such as patient care, supportive therapy, and integration with pharmacological cancer treatments. These are as fundamental to the discipline as the technical aspects and represent the second essential pillar of radiation oncology.

## Forces driving the increased use of AI automation in radiation oncology

Artificial intelligence automation in treatment planning might significantly improve patient outcomes in radiation oncology. Widespread implementation of adaptive radiotherapy, including MR-guided adaptive radiotherapy, is only possible with AI automation to reduce on-table time to a reasonable duration [3, 4]. Same-day treatment, with a short interval between planning imaging and treatment delivery, is technically feasible with AI automation and has the potential to enhance patient experience and outcomes in radiation oncology. In addition, tumors grow from diagnosis to the start of treatment, thus worsening the treatment outcome [7]. This critical time period could be greatly reduced with AI automation. Moreover, emerging and third-world countries have a lack of radiotherapy specialists, which affects millions of patients. AI automated planning is already employed in efforts like the Radiation Planning Assistant (RPA) that aim to increase global access to radiation therapy [8]. Finally, AI automation could enable more complex treatments that are prohibitively time consuming today. Examples include more sophisticated target volume definitions, sparing of an extended set of risk structures, and local treatment of a large number of targets in patients with metastatic disease.

The demographic crisis will be an important megatrend in the western world and likely an important driving force for increased use of AI automation in radiation oncology as well. As the number of older people in western populations increases, the number of required radiation therapy treatments will also rise [9, 10]. At the same time, the share of younger people for the radiation therapy workforce will decrease, creating an enormous supply gap in the context of a pre-existing workforce crisis [11, 12]. Artificial intelligence automation is an obvious answer to the required increase in productivity and, given the scope of the challenge, resistance to an increasing role of AI in radiation oncology seems to have little promise of success. It is, however, essential to maintain medical oversight and control. Therefore, determining the optimal interaction between AI and radiotherapy experts, e.g., through interactive AI models and workflows [13], will be crucial for the future of radiotherapy treatment planning.

## Making AI expertise an integral part of radiation oncology

If AI in radiation oncology is left to external entities (i.e., MedTech companies or the fields of computer sciences and engineering), the role of radiation oncologists, medical physicists, and the entire field bears the risk of being diminished as much as the role of AI will grow. Radiation oncology could be stripped of its defining core competency. Thought through to its end, radiation treatments could become completely autonomous, supervised and applied by any oncology discipline.

To avoid such a fate and to make the most out of the combination of AI and radiation oncology, AI expertise should become an integral competency of our field. In that way, the field of radiation oncology will grow with the increasing role of AI in medicine. A part of the solution could be to integrate computer scientists and AI experts into radiation oncology. However, this will be limited by the availability of computer science experts and the fact that here, AI and radiation oncology expertise remain separated across different professions. Therefore, we should raise the technical competency of medical physicists and radiation oncologists as functional units of our field to become experts for AI in radiation oncology. If AI is to become an integral part of radiation oncology, then AI expertise has to become an integral part of our competence as well. Increased AI and computer science skills could also allow for a much more differentiated application of AI methods in radiation oncology, tailored to the individual patient and center. However, the integration of radiation oncology and AI expertise within medical physicists and radiation oncologists is particularly important to identify the future directions of our field and to spark ideas on the optimal implementation of AI technologies within radiation oncology. Raising the AI competency within radiation oncology will also ensure that the driving intellectual forces that will shape the future of radiotherapy will emerge from within our field and not from outside.

## Why integrating AI expertise is crucial for future innovations in radiation oncology

In 1865, the French physiologist and physician Claude Bernard famously called the laboratory “*the true sanctuary [sanctuaire] of medical science*,” while he considered the hospital to be “*merely […] the vestibule of scientific medicine; […] the first field of observation into which the physician must enter*” [14]. While today we can certainly argue that AI research belongs as much to the inner heart of academic radiation oncology as does laboratory research, the translational imagery of Bernard is more relevant for the point to be made. Bernard does not intend to diminish the role of clinical medicine by calling it “*vestibule*”; instead, he intends to illustrate the necessary integration of clinical expertise with the laboratory methodology within the individual physician: “*The physician, […], must, upon leaving the hospital, go to their laboratory, and it is there that they will seek, through experiments […] to make sense of what they have observed in their patients […] It is there, in a word, that they will create true medical science. Every learned physician must therefore have a physiological laboratory […]*” [14].

For AI in radiation oncology, similar to the introduction of the laboratory method into medicine, the methodology cannot be separated from its field of application. It is in individual physicians and physicists within radiation oncology that the methodological possibilities of AI and the clinical needs and opportunities can be brought together most effectively. Practical experience in AI development and application combined with the practical knowledge of radiation oncology is therefore what will bring forth the most promising new concepts and abstract ideas for the future of this field.

The necessity for a deep integration of AI expertise into radiation oncology is not only suggested by the history of scientific medicine but also by the experiences in the computer industry as well: “*My observation is that the doers are the major thinkers. […] And if we really go back and we examine: Did Leonardo [da Vinci] have a guy off to the side that was thinking 5 years out into the future, what he would paint or the technology he would use to paint it? Of course not. Leonardo was the artist, but he also mixed all his own paints, he also was a fairly good chemist: He knew about pigments, knew about human anatomy. And combining all of those skills together […] was what was resulted in the exceptional result. And there is no difference in our industry […]*” (from The Machine that Changed the World, Steve Jobs 1990 [15]).

## How to strategically increase AI proficiency within radiation oncology

If integrating AI expertise is identified as a strategic aim for the field of radiation oncology, we need to think about how to achieve it. Scientific societies like the DEGRO, ÖGRO, SASRO, and ESTRO could play a key role by offering certified programs for obtaining AI expertise for radiation oncologists and medical physicists at different levels of competencies. Certified AI competency levels from leading scientific societies (e.g., in the form of a suffix that can be placed behind one’s name) could create a large motivation for participation and for increasing AI expertise in the field. This also implies that the education of future radiation oncologists will become significantly more complex. Core competencies in the principles of deep learning applications, imaging technologies like MRI, and image computing methods (e.g., non-rigid image registration) need to be integrated into the training and examination of radiation oncology residents and medical physicists, as the proficient application of these technologies is important for clinical practice. To address these demands, it may be necessary to establish inter-institutional courses that provide comprehensive training across these competencies.

## AI as the continuation of radiation oncology with new means

Parallels to the current relationship between AI and medicine can also be drawn from the philosophy of Clausewitz. Clausewitz famously demanded the primacy of policy, criticizing the rigid separation between the military and political domains of his time [16]. In one of his key findings, he recognized that the “*[…] means can never be considered in isolation from their purpose […]*” (“*niemals kann das Mittel ohne Zweck gedacht werden*”) [16]. For AI in radiation oncology, the primacy of medicine should similarly be postulated. AI should be regarded as a tool of medicine, not different from other technologies adopted by medicine throughout the ages as its own. For the purposes of radiotherapy, the development and application of AI technology therefore belongs at least as much to radiation oncology as it does to computer sciences or engineering. At the same time, clinical experts within radiation oncology require computer science knowledge, as the attainable medical objective is also shaped by the technical possibilities (“*[Der Zweck] muss sich der Natur des Mittels fügen und wird dadurch oft ganz verändert, aber immer ist er das, was zuerst in Erwägung gezogen werden muß*.”) [16]. AI should therefore be incorporated into radiation oncology as a novel medical tool that serves a clinical therapeutic purpose. As such, AI should not be seen as the end but rather as the continuation of radiation oncology with new means.
